# Supplementary material for: Are carbon emissions trading and green financial instruments synergistic? -Comprehensive quantitative research based on content analysis
Source: PLoS One. 2024 Mar 7;19(3):e0298601. doi: 10.1371/journal.pone.0298601 (PMC10919601; doi:10.1371/journal.pone.0298601)
Supplement: S1 Data — (ZIP) [file pone.0298601.s001.zip › Data/地级市Z_F工作报告.zip!/╣π╢1⁄2/╓Θ║ú╩╨/└┤╫╘╣1⁄2╓┌║┼í╛┬φ┐╦╩2╛▌═°í┐_2006.docx]

各位代表：

　　现在，我代表市人民政府向大会作政府工作报告，请予审议，并请市政协各位委员和其他列席人员提出意见。

　　一、“十五”期间和２００５年工作回顾

　　“十五”期间，市政府在省委、省政府和市委正确领导下，在市人大、市政协监督支持下，坚持以邓小平理论和“三个代表”重要思想为指导，树立和落实科学发展观，狠抓发展第一要务，带领全市人民扎实推进经济建设、政治建设、文化建设和社会建设，全面完成了“十五”计划提出的各项任务，全市经济社会持续快速协调健康发展。

　　五年来，地方生产总值年均增长１３．８％，财政收入年均增长１５％；２００５年实现的工业总产值相当于“九五”期末的两倍。经济结构调整优化，区域发展不平衡逐步改善，工业重型化、高级化趋势明显。高新技术产品产值占工业总产值的３８％，比“九五”期末提高了１４．５个百分点。一二三产业结构由２０００年的４．３：５２．２：４３．５调整为３．２：５１．９：４４．９。体制改革不断推进，政府公共服务职能加强，依法行政水平提高。对外开放迈上新台阶，利用外资和外贸规模显著扩大。重大项目建设取得一系列突破性进展，城市基础设施不断完善。生态环境保护成绩突出，建设国家级“生态示范区”，连续四年获得全省环保责任考核第一名。连续六年被评为“国家科技进步先进城市”，基础教育和高等教育实现跨越式发展，建设“广东信息化综合试点城市”，文化、卫生、体育等各项社会事业蓬勃发展，城乡居民生活水平逐步提高。“十五”时期，是珠海适应环境变化、应对各种挑战，取得重大发展成就的五年；是人民群众从改革和发展中得到更多利益和实惠的五年；也是承前启后、为珠海“十一五”时期更快更好发展奠定良好基础的五年。

　　２００５年是实施“十五”计划的最后一年，也是建立珠海经济特区２５周年。一年来，市政府带领全市人民，发扬特区创新精神和求实作风，深入学习贯彻党的十六届四中、五中全会和温家宝总理视察珠海重要讲话精神，认真落实中央宏观调控政策，按照省委、省政府和市委一系列战略决策及重要工作部署，积极应对能源供应紧张、西江百年一遇特大洪水、高致病性禽流感疫情、特大咸潮等不利因素带来的影响，围绕“加快发展，提高质量，化解矛盾，改善民生，优化环境，依法行政”的总体思路做好政府各方面的工作，圆满完成市六届人大二次会议部署的任务，全市经济和社会发展取得了可喜的成绩。

　　（一）经济保持较快发展

　　全年地方生产总值６３４．６亿元，同比增长１３．１％。工业增加值３２６．１亿元，增长１５％；工业总产值１６１０．５亿元，增长１４．５％。农业和农村经济结构继续调整，全市农林牧渔业总产值３３．８亿元，增长６．４％。投资、消费、出口出现多年未有的全面增长局面。固定资产投资总额２１６．３亿元，增长２０．３％。房地产、汽车、文化娱乐等成为新的消费增长点，社会消费品零售总额２２０．１亿元，增长１６．３％。旅游业平稳增长，接待过夜游客６４０．２万人次，增长１４．１％。港口货物吞吐量３５５６．９万吨，增长１１％。外贸规模创历史新高，进出口总额２５７．３亿美元，增长１８％，其中出口额１０７．７亿美元，增长１９．２％。财政一般预算收入４８．９７亿元，增长２１．４％，是近四年来的最高增幅。在珠海产生的各种税收总收入１６９．６亿元，增长１３．８％。金融机构存款余额突破千亿元大关，年末全市金融机构本外币存款余额１０１４亿元，比年初增长１９．１％；本外币贷款余额４８６．７亿元，比年初增长７．５％。

　　（二）经济结构调整取得成效

　　高新技术产业保持快速发展，高新技术产品产值达６１２亿元，增长２９％。全市有１项科技成果获得国家科技进步奖，８项科技成果获得省科学技术奖，国家和省级名牌增加到４３个。公共技术服务平台建设取得成效，建成珠海南方集成电路设计服务中心。高新技术企业发展壮大，世界首台超低温数码热泵多联空调机组在格力电器顺利下线，珠海炬力成功在美国纳斯达克上市。外源型经济快速发展，利用外资质量提高，大项目增多，新批外商直接投资项目５８７个，其中，１０００万美元以上的项目１０４个，世界５００强企业增资和新投资项目１６个；实际利用外资６．７亿美元，增长４１．７％。内源型经济发展加快，国有企业改革深化，上市公司股权分置改革顺利推进，市属国有企业实现利润总额近１２亿元。民营经济发展加快，民营企业税收贡献率提高，在扩大就业、外贸出口、科技创新等方面发挥重要作用。

　　（三）重大项目建设取得重要突破

　　粤西沿海高速公路珠海段和机场北路竣工通车。广珠城际轨道动工建设。江珠高速公路、两个５万吨级集装箱码头、石化公用码头、洪湾港一期和南横路等交通项目加紧施工。广珠铁路纳入省部合作项目，即将复工。港珠澳大桥前期论证取得重要进展。珠海电厂３、４号机组建设进展顺利。洪湾电厂完成改造，产能扩大到４５万千瓦。拱北２２０千伏变电站投产运行。中海油天然气终端项目建成投产。ＬＮＧ接收站项目落户珠海。珠澳跨境工业区即将申报国家验收投入运作。格力电器五期等一批重点工业项目投产，大型旅游项目海泉湾度假城开业。

　　（四）统筹资源化解矛盾工作进展良好

　　严格管理财政资金，积极培植税源，税收征管和债权追收工作进一步加强。珍惜使用土地资源，规划和国土行政管理体制改革顺利实施，规划经费得到保障落实，各层次的城市规划加快编制。制定土地管理领导小组议事办法等规章制度。全年依法收回不按期开发用地９．９平方公里。土地储备力度加大，储备总量达５９．９平方公里。土地明显升值，全年市级土地收入达２３．０６亿元。公共资源经营管理加强。公用事业特许经营改革取得实质性突破，城市管道燃气特许经营改革正式实施。出台《珠海市建设工程招标投标管理办法》，规范招标投标管理。全面清理机关和事业单位经营性资产，公务用车制度改革顺利实施，车辆经费比上年节省１４００多万元。多方筹集还款资金，积极有序清偿债务，已解决政府工程欠款合计１３．２亿元。农信社改革顺利推进，珠海商业银行重组和珠光集团债务重组工作取得了积极进展。

　　（五）政府公共服务和社会管理职能得到加强

　　规范审批行为，完成第三轮行政审批制度改革。教育事业发展迈上新台阶。香洲区、金湾区进入省级“教育强区”行列。市属三家医院改扩建工程进展顺利。农村合作医疗保障能力逐步增强，海岛医疗卫生条件得到改善。有效防控禽流感疫情。文化、体育事业蓬勃发展，首届全国教师合唱节、南方文化产业论坛等大型活动获得成功。有线数字电视整体转换工作启动，市图书馆续建改造完成。生态保护和污染防治工作继续加强，成为“国家环保模范城市”免检城市。加强路面、社区防控，打击“两抢一盗”等违法犯罪活动见成效。安全生产专项治理力度加大，对重大消防隐患单位挂牌督办，全年安全生产形势平稳。整治室内违法装饰装修取得初步成效。连续第二年获全省实施城乡防灾减灾工程优秀奖励。连续第三年获得全省人口与计生目标管理责任制综合考核第一名。拱北口岸电子验放系统和旅客自助查验系统投入使用，口岸通关能力进一步提高。打击走私保持高压态势。在地统计管理体制改革全面实施，完成经济普查任务。双拥共建和人防工作成绩突出，获得“全省人民防空先进城市”称号。自觉接受人大法律监督、政协民主监督和广泛的社会监督。认真办理市人大议案、代表建议和政协提案，大力推行政务公开制度，机关作风建设深入开展。政府法制工作成绩显著，普法、人民调解、法律援助工作取得成效。实施“助残工程”，１．５万名残疾人受惠。精神文明建设成效显著，再次获得“省文明城市”称号。新闻出版、民族宗教、外事、侨务、档案、气象、仲裁、地方志、港澳流动渔民工作取得新的成绩。积极支持工会、共青团、妇联和工商联、科协、文联、侨联、残联、社科联等发挥职能作用，促进了社会协调发展。

　　（六）人民生活水平进一步提高

　　全年城镇居民人均可支配收入１．８９万元，按可比口径增长７．９％；农渔民人均纯收入６５５０元，增长８．３％。城乡居民本外币储蓄存款余额５１３．７亿元，同比增加１４．１％。全市新增就业岗位近５万个，城镇登记失业率为２．７９％。实施“百万农村青年技能培训工程”和“百企扶百村就业工程”，参加培训人员达２．８万人，实现转移就业的农村富余劳动力超过１万人。社会救助体系不断完善，“扶贫济困”取得成效。社会保障制度改革有新突破，出台《珠海市社会养老保险条例》、《珠海市农民和被征地农民养老保险过渡办法》和《珠海市社会保险反欺诈办法》。社保基金和参保人数稳步增长，保险基金征缴率达９９．４％。食品药品安全监督力度加大，豆制品安全专项整治取得成效，无重大食品药品安全事故发生。住房公积金稳健运作，红旗、平沙危房改造加快实施，为城镇住房特困户发放租房补贴。农村公路网逐步完善，农村生活环境改善。老龄工作、慈善等事业有新进步。

　　各位代表！“十五”时期和２００５年市政府工作取得的成绩，是全市人民团结奋斗、扎实工作的结果；离不开中央、省驻珠单位和驻珠军警部队的大力支持；也与历届市委、市政府打下的良好基础分不开。在此，我代表市人民政府，向为珠海改革开放和现代化建设付出辛劳、作出贡献的全体市民表示崇高的敬意！向所有关心支持珠海发展的港澳台同胞、海外侨胞和社会各界朋友表示衷心的感谢！

　　在肯定成绩的同时，我们也看到珠海在发展中存在诸多矛盾和问题。主要是：经济总量偏小、产业发展不充分仍是制约珠海经济社会发展最根本的矛盾，经济结构不优、发展不平衡的问题仍相当突出；自主创新能力不足，大部分产业处于产业增值链的低端，经济发展总体上仍未摆脱低质量、低效益的粗放式增长模式；经济社会发展面临着体制性、机制性障碍，改革攻坚任务仍然繁重；未来几年，化解矛盾、处理历史遗留问题的任务仍十分艰巨；资源紧缺和生态环境压力对城市可持续发展带来的挑战日益加剧；政府在社会管理和公共服务方面存在薄弱环节，就业、教育、卫生、环保、治安、公共安全、城市道路交通等方面一些关系群众切身利益的问题亟待解决；公务员队伍建设有待继续加强，一些工作人员工作“不在状态”，工作作风有待改进。对这些问题，我们要清醒认识，客观面对，在改革和发展中采取得力措施，努力加以解决。

　　二、“十一五”时期发展展望

　　综观“十一五”时期国内外发展大势，珠海既面临难得的发展机遇，又面临严峻的挑战，但总体上机遇大于挑战，珠海将迎来新一轮跨越式发展的战略机遇期，这也是我市全面建设小康社会、率先基本实现社会主义现代化的关键时期。

　　“十一五”时期，我们要坚持以邓小平理论和“三个代表”重要思想为指导，以科学发展观统揽全局，以建设珠三角区域中心城市和实践科学发展观先行示范市为目标，着力推动珠海经济社会转入科学发展、和谐发展轨道，坚持以人为本，速度与效益并重，发展经济与保护环境双赢，科学规划、合理布局，城乡区域协调发展五大原则，大力推进发展模式、体制机制、对外开放、科技自主、城市建设与管理五大创新，发挥区位、环境、特区品牌三大优势，全面实施“工业强市”、“科教兴市”、“三产旺市”三大战略，把珠海打造成为适宜居住、适宜创业、富有魅力的特色城市。

　　“十一五”时期珠海经济社会发展的主要目标是：保持经济持续快速健康发展，力争到２０１０年率先基本实现社会主义现代化；本地生产总值前两年平均增长１３％以上，后三年平均增长１５％以上，预期２０１０年比２００５年翻一番，人均ＧＤＰ超过１万美元；经济增长方式明显转变，资源利用效率显著提高，单位生产总值能耗比“十五”期末降低１５％以上；自主创新能力大幅提高，形成一批具有自主知识产权和知名品牌的优势企业；改革开放和区域合作取得新突破，社会主义市场经济体制更加健全；生态环境保持良好；创建“教育强市”、“文明城市”工作有新突破，社会保障体系比较完善，城乡居民收入明显提高，人民生活总体上达到富裕的小康水平。

　　各位代表，市政府根据《中共珠海市委关于制定珠海市国民经济和社会发展第十一个五年规划的建议》组织编制的《珠海市国民经济和社会发展第十一个五年规划纲要（草案）》提交本次大会审议。纲要提出了“十一五”期间十项主要任务：一是壮大经济规模；二是加快基础设施建设；三是打造城市新格局；四是提高自主创新能力；五是建设社会主义新农村；六是全面推进综合配套改革；七是进一步扩大开放；八是建设资源节约型和环境友好型城市；九是加强精神文明建设；十是促进社会和谐发展。今后五年，市政府要团结带领全市人民，围绕“十一五”规划目标和主要任务，切实抓好各项工作措施的落实，充分调动各方面的积极性、主动性和创造性，聚精会神搞建设，一心一意谋发展，转变发展理念，创新发展模式，突破发展瓶颈，提高发展质量，增强发展后劲，励精图治，锐意进取，开拓创新，为高质量、高水平实施“十一五”规划而艰苦奋斗。同时，我们还要坚持不懈地加强政府自身建设，着力推动政府职能转变，努力建成依法行政、高效清廉的服务型政府。

　　三、２００６年的主要工作任务

　　２００６年是实施“十一五”规划的开局之年，市政府工作的指导思想是：以邓小平理论和“三个代表”重要思想为指导，坚持以科学发展观统领经济社会发展全局，认真贯彻中央和省的各项工作部署，抓住新型工业化这条主线，加快产业聚集，积极转变经济增长方式，努力增强自主创新能力，推动经济“又快又好”发展；突出抓好重大项目建设，加强城市规划建设管理，深化改革，扩大开放，统筹城乡发展，致力改善民生，建设依法行政的服务型政府，全力推动珠海“三个文明”与和谐社会建设，为“十一五”规划实施开好头、起好步。

　　２００６年我市经济社会发展的主要预期目标是：地方生产总值增长１３％，每万元生产总值能耗下降２％，工业增加值增长１６％，财政一般预算收入增长１２％，社会消费品零售总额增长１４％，固定资产投资增长１５％，外贸出口增长１２％，居民消费价格总指数上升２％左右，实际利用外商直接投资额增长１０％，引进内资注册资本增长２０％，城镇登记失业率控制在３％以下，城镇居民人均年可支配收入增长６％，农渔民人均年纯收入增长７％。

　　为实现以上目标，要重点做好以下工作：

　　（一）加快发展，壮大经济总量

　　坚持“大办工业”，注重“办大工业”。坚持把推进新型工业化作为全市经济工作的主线，集中精力、集中资源支持工业发展。充分发挥我市在区位、资源、市场等方面具备的比较优势，着力抓好科学规划、政策引导、硬件支撑、环境优化和载体建设等关键环节，顺应国际产业转移趋势，抢抓我省产业向重型化、高级化方向调整升级带来的机遇，努力承接符合我市发展规划的产业转移，加快产业聚集，壮大产业规模。

　　抓好“两个亮点”开发建设。临港工业区和横琴经济开发区是我市下一步发展的两个亮点。进一步从战略上重视临港工业区发展，今年要加快东大堤和石化基地、装备制造专区填土工程等基础设施建设，狠抓重点项目的落实，努力在发展临港型重化工业方面有更大的作为。积极主动加强与上级沟通，推动泛珠三角横琴经济合作区规划与我市相关规划的衔接，积极配合国家和省做好开发前期准备工作，争取已签约并符合总体规划的重点项目早日动工建设。

　　抓好龙头项目引进和建设。大型龙头项目是产业发展的支撑，珠海要靠引进大项目打牢产业基础，重大项目对珠海发展至关重要。我们要付出更大的努力，争取把可能的机会变为现实。要进一步强化项目配套服务，推动ＰＴＡ二期、华丰造纸等一批大型工业项目加快建设，力促已落实的龙头项目早动工、早投产。

　　继续坚持实施外向带动。发展外向型经济是珠海的优势。继续扩大利用外资，提高招商引资水平。创新招商方式，强化产业招商，鼓励企业增资扩产。支持出口型企业发展。优化外贸市场结构，开拓新兴市场，积极发展服务贸易。引导出口加工型企业提高增值率，促进加工贸易转型升级，增加就业和对本地财税等方面的贡献。加快横琴口岸新联检楼建设，完善拱北口岸“一站式”电子验放系统和旅客自助查验系统建设，进一步提高通关能力。

　　发挥投资拉动作用。去年市政府部分投资项目进展不理想，今年要总结经验教训，切实提高项目组织管理水平，保持适度的政府投资力度。政府投资今年继续重点投向教育、卫生、市政、文化、体育、水利等公共事业领域。严格执行《珠海市政府投资项目管理条例》，推进政府投资建设工程组织实施方式改革，提高投资效益。与金融机构加强协调，用好更新改造财政贴息资金，鼓励企业加大更新改造投资。加快投融资体制改革，消除投资领域不合理的政策障碍，充分发挥各种社会投资主体的积极性。加大对内招商力度，建立对内招商与对外招商同等考核和奖励的制度，吸引内地大中型国有企业、上市公司和有实力的民营企业到珠海投资。

　　大力发展现代服务业。重点扶持为产业提供直接服务的商贸、物流、金融等服务业的发展。引导商业合理布局，建设特色商业街区，在西部地区规划建设大型商业设施。加快建设大型会展设施，积极发展会展业。扶持总部经济发展。优化消费环境，培育新的消费热点。开拓农村消费市场，扩大农村消费。进一步重视旅游业发展，树立大旅游概念，高标准做好旅游产业发展规划，加强旅游行业管理和区域旅游合作，加快大型旅游景区和设施建设，大力推进自然生态、人文历史与旅游文化相结合，提升旅游品位，丰富珠海旅游城市的内涵。对房地产业进行科学调控、有效监管和合理引导，推动房地产业健康有序发展。办好第六届中国航展，提高办展水平和综合效益。

　　（二）科学发展，提高经济发展质量

　　努力建设创新型城市。提高自主创新能力是我市转变经济增长方式、提高经济质量的关键。认真贯彻落实全国科技大会精神，坚持“自主创新、重点跨越、支撑发展、引领未来”的指导方针，全面实施自主创新战略。重点通过财税支持、金融扶持、政府采购、科技计划、公共技术平台建设、知识产权保护等方面的政策措施，加快建设以企业为主体、以市场为导向、产学研相结合的技术创新体系。进一步发挥珠海大学园区高校和科研机构的作用，鼓励多种形式的产学研合作。加强人才工作，用好专项人才开发资金，抓好人才引进和培养，壮大人才队伍。

　　着力提升产业质量。围绕核心技术和骨干企业，有重点地发展电子信息、光机电一体化、新材料、新能源、航空修造等高新技术产业。继续支持信息产业特别是软件和集成电路产业的发展，走信息化带动新型工业化道路。制定建设中医药强市实施意见，发展中医药产业，扶持制药企业做大做强，为广东建设中医药大省作出贡献。制定优惠政策，扶持动漫等文化创意产业发展。广泛运用高新技术和先进适用技术改造提升传统制造业，逐步改变低附加值的低端产业格局。

　　优化企业组织结构。加强中小企业服务，政府牵线搭桥，建立大型龙头企业与中小企业产业协作配套平台，引导大中小型企业形成相互配套、专业分工的产业发展格局。实施品牌战略，引导、鼓励和支持企业积极开展自主品牌经营。继续对创出全国、全省知名品牌的企业实行重奖。

　　提高园区开发水平。多渠道筹措资金，完善产业园区基础设施和第三产业配套，优化发展环境。按照产业集群发展要求，进一步明确园区功能定位，制定园区产业发展指导目录和产业规划布局要点，引导产业合理布局，推动特色产业发展壮大，增强园区产业竞争力。

　　建设节约型社会。按照“减量化、再利用、资源化”三大原则，提高资源节约和再利用水平，发展循环经济。政府今年安排专项资金，支持推广应用新技术、新工艺和新材料，淘汰能耗高、效率低、污染严重的技术和设备，鼓励企业实施清洁生产、循环式生产，提高煤、电、油、气等能源利用效率。加强节能管理，推广节能技术。重视推动再生水使用，节约水资源。政府机构要带头节约，推广绿色消费，倡导全社会形成节约风尚。

　　（三）突破发展瓶颈制约，继续狠抓重大基础设施建设

　　大力推进交通基础设施建设。加快完善陆路综合交通网络。全力促成广珠铁路年内全线复工。支持推动广珠城际轨道、江珠高速、粤西沿海高速支线等项目建设。加紧建设南横路、情侣北路，分段改造国道Ｇ１０５线珠海段、省道Ｓ２７２线珠海段、省道Ｓ２７０线和省道Ｓ３６６线快速干道，提高珠海东西向主通道通行能力。继续配合推进港珠澳大桥建设前期工作。

　　增强双港功能。加快推进珠港机场合作，盘活珠海机场资源，完善机场与周边联系的路网，有效发挥空港作用。以建设沿海主枢纽港为目标，处理好工业发展与港口发展的关系，控制好、开发好宝贵的岸线资源。抓好续建高栏港区两个５万吨级集装箱码头、中化格力８万吨级公用石化码头、洪湾码头一期等港口工程。实施高栏公共进港航道二期扩建工程。抓紧筹建高栏港区１０万吨级煤码头、１５万吨级矿石码头、桂山１０万吨级油码头改造、南水作业区大突堤等项目，积极推动高栏港区４个１０万吨级集装箱码头、３０万吨级油码头和第二防波堤等工程项目的前期工作。抓好万山港区成品油码头和３０万吨级原油码头规划等相关工作。着手开展高栏港各作业区之间联接方案研究。

　　加快大型能源项目建设。协调珠海电厂和深能洪湾电厂新建机组并网发电。加快２２０千伏八一输变电工程建设，建成５００千伏珠海电厂－香山变电站工程，做好５００千伏珠海输变电工程前期工作。支持规划建设珠海电厂５、６号机组等能源项目。进一步抓好广东高栏ＬＮＧ项目、南海天然气横琴接收站建设工作。加快城市燃气管网建设，让更多的居民使用上管道燃气。

　　抓紧实施水资源调控重点工程。推进水资源安全体系建设，积极应对咸潮威胁，加快实施咸期应急供水工程，今年内要完成工程总量的８０％，争取明年投入使用，从根本上增强城市供水保障能力。加快推进分质供水。加紧建设乾务水厂、南区水厂、黄杨泵站等供水工程项目，系统推进西部农村水改工程，争取明年内让农村群众全部喝上符合国家标准的自来水。继续建设白蕉联围海堤、小林联围鸡啼门下段海堤和联合水闸、乾务赤坎大联围平沙东堤和莲洲堤段及三前水闸等重大防洪减灾水利工程，提高城市防洪能力。

　　切实做好重大项目组织管理工作。形成“动工建设一批，前期推进一批，规划培育一批”的重大项目工作机制。实施重大项目工作责任制，明确职责分工，加强协调配合，解决好重大项目征地、拆迁、环保、交通、供水、供电和建设过程中遇到的实际问题。加强重大项目建设工作的监督检查，保障重大项目工程质量和施工安全。

　　（四）增强可持续发展能力，加强城市规划建设管理

　　从战略上重视城市规划。珠海面临重大发展机遇，应更加突出规划的龙头地位，充分发挥规划对城市经济社会发展的先导、主导和统筹作用。新一轮城市规划要与“十一五”经济社会发展规划紧密衔接。继续增加规划经费，加快完成《珠海城市空间发展战略规划（２００６－２０３０）》、《珠海市近期建设规划（２００６－２０１０）》，以及斗门区、临港、南湾、唐家湾地区等分区规划，尽快实现近期建设地区的控制性详细规划全覆盖。着手进行西部沿海经济带发展整体规划。推行城市规划公示制度，提高规划的公开性和透明度。加强规划立法和实施监督工作，确保城市规划实施的有效性、严肃性和延续性。

　　打造城市建设新亮点。认真实施城市绿地系统规划，今年要重点建设海天公园、凤凰山森林公园等一批公园和绿地。综合整治香洲渔港，推进前山河清淤工程和沿岸整体改造，打造新的城市景观带。积极盘活“烂尾楼”。加大市政建设投入，进行主城区市政道路、园林、路灯设施改造升级工程，在主城区建设１２条林荫大道，在繁忙路口加紧规划建设人行天桥或地下通道。落实公交优先原则，完善公交基础设施，加快规划建设一批立交桥、停车场设施，优化城市路网结构，保障城市交通安全畅顺。继续推进香洲辖区城中旧村改建，市政府原承诺的政策以及给予村（居）民和开发商的优惠不变，确定的原则不变。

　　理顺城市管理体制。加快调整和完善城市管理体制，明确市、区政府城市管理职责分工，充分发挥市专业部门的职能作用和区级政府的积极性，探索专业管理相对集中、综合管理重心下移的城市管理新模式。推动市政设施养护市场化步伐，提高市政设施管养标准和水平。推进相对集中行政处罚权工作，建立健全城市管理快速反应机制，推动城市管理网格化、智能化建设，增强城市管理效能。加大城市管理监督力度，大力查处违章建筑，整治非法室内装饰装修行为。加强小区物业管理，依法维护业主和物业管理公司合法权益，构建和谐社区。

　　管好用好土地。按照“控制增量、管住总量、消化存量、提高质量”的要求，把好项目准入关，提高土地单位面积投资强度，促进节约集约用地。完成市级土地利用总体规划的修编和镇级土地利用总体规划编制。加强土地储备，加大土地市场调控力度，实施《珠海市国有土地闲置费征收实施办法》和《珠海市土地使用费征收办法》，盘活存量土地资产，为解决历史遗留问题和重大项目建设筹措资金。依法严格保护好耕地和林地。加强土地管理执法队伍建设。

　　抓好生态年建设。把今年作为“生态建设年”，全面实施《珠海生态市建设规划》，启动“国家生态园林城市”创建工作，进一步提升珠海整体环境质量。重点抓好十项环境工程，包括：主城区污水管网改造及排洪渠整治工程，前山河流域污水集中处理工程，南区污水处理厂工程，井岸生活污水处理厂工程，三灶污水处理工程，西坑尾垃圾填埋场及渗滤液处理工程，西部地区生活垃圾填埋场工程，医疗废物和建筑垃圾处理工程，珠海发电厂１、２号机组脱硫工程和生态示范村创建工程。加强环保能力建设，完善环境监测和预警体系，构建环境污染事故应急处置机制。加强近岸海域和海岛生态环境综合治理。通过生态年建设活动，确保全年环境空气质量优良率达到９５％以上，城市生活污水处理率超过７０％，城市生活垃圾无害化处理率达到８０％以上，西部地区和农村环境得到进一步改善。

　　（五）增强发展动力，继续深化改革和扩大开放

　　改革高新区和临港工业区管理体制。针对两区现行体制运行中出现的矛盾和问题，适应两区新的发展目标，通过新一轮改革，形成责任主体明确、权力界定清晰、办事方便快捷、利益分配合理的管理体制，更好地调动各方面的积极性，形成发展的合力。

　　深化国有企业和国有资产监管管理体制改革。完善国有资产出资人制度，确保国有资产监管机构依法履行出资人职责。探索市属国有资本经营预算制度，进一步完善收益管理制度。建立健全国有资本有进有退、合理流动机制，推动国有资本向基础性、公用性和重点关键领域集中，加大引进战略投资力度，逐步实现产权多元化。完成市属国有控股上市公司股权分置改革，加快推进公共交通和城市供水等政府公共资源特许经营改革。建立健全非经营性资产的监管体制，防止国有资产流失。

　　发展壮大民营经济。认真落实省、市扶持民营经济发展的政策措施，民营企业与其它企业一视同仁，平等待遇。支持民营资本进入法律法规未禁止的一切行业和领域，凡是对外资开放的，也对民营资本开放。鼓励民营资本参与国有企业改组改造。拓宽民营企业融资渠道，引导民间融资规范发展。坚持一手抓扶持服务，一手抓规范提高，引导民营企业实现产业升级和管理制度创新。

　　完善社会主义市场经济体系。整顿和规范市场经济秩序，严厉打击传销和变相传销、偷逃骗税、逃汇骗汇等违法犯罪活动，保持打击走私高压态势。政府带头树立诚信形象，推动全社会信用体系建设。发展具有区域辐射力的产权交易市场，加大对各类有形要素市场的规范监管力度。加快现代金融产业发展，鼓励银行、证券、保险、基金以及信用担保、风险投资机构进入珠海。继续支持农信社和商业银行改革，努力维护金融稳定。规范发展行业协会和中介组织，进一步健全现代市场体系。

　　深化财政体制改革。探索建立社会综合治税工作体系，加强税收征管，依法征税。实施《珠海市非税收入管理条例》，将非税收入纳入财政体系管理。按照公共财政的要求，优化支出结构，增加公共服务支出，支持农村经济和社会事业的发展。进一步完善市、区两级激励型财政管理体制。继续深化部门预算、国库集中支付和政府采购制度改革，积极建立财政绩效评价制度，加快金财工程建设，提高科学理财水平。

　　提高对外开放水平。继续发挥经济特区“窗口”和“试验田”作用，走改革促开放、开放促改革的路子。进一步优化体制、制度和政策环境，使政府运行更加符合世贸规则，努力率先建成符合国际通行规则的制度环境。在继续抓引进的同时，切实加强消化吸收和创新提高。支持有条件的企业“走出去”。

　　积极参与区域合作。把握角色和定位，在泛珠三角区域合作中积极发挥作用。加强与珠三角周边城市在规划、产业、基础设施、环保等领域的合作，努力实现优势互补，互动发展。扩大珠港澳合作，加快珠澳跨境工业区建设，扩大珠港澳在旅游会展、跨境基础设施、口岸通关、现代物流和金融业等方面的合作。积极拓宽对台经济技术交流与合作领域。

　　（六）统筹城乡发展，扎实推进社会主义新农村建设

　　以工促农、以城带乡。要按照“多予少取放活”的方针，加快推进我市农村工业化、城镇化和农业产业化，启动强镇富民、安居乐业、生态文明、扶贫济困和固本强基“五项工程”，加快建设生产发展、生活宽裕、乡风文明、村容整洁、管理民主的社会主义新农村，形成城乡协调发展格局。

　　增强区镇经济实力。香洲区要加快产业升级步伐，重点发展现代服务业和高增值的制造业。金湾区、斗门区重点推进新型工业化，同时加快规划建设新城镇，使工业化与城市化协调发展。万山区拥有关系我市长远发展的重要战略性资源，要积极开展深水岸线资源开发研究，在保护好资源的前提下，加大海岛基础设施建设力度，有重点地发展海岛旅游、中转仓储和海洋渔业，提高渔民收入水平。各镇都要结合自身特点，有重点地发展特色经济。进一步发展壮大农村集体经济。

　　加快农村经济发展。用抓工业的理念来抓农业产业化，实施农业园区和农业龙头企业带动战略，规划建设农科中心等一批重点农业产业园区，着力改善农业生产条件，建立新型农村科技推广服务体系，健全农产品流通网络，提高农业综合效益。加强农田水利和农村基础设施建设。继续实施“农村青年技能培训工程”、“百企扶百村就业工程”，加强农民就业技能培训，促进农村富余劳动力向第二、三产业转移就业。积极运用市场机制，推进集体建设用地使用权流转，允许农村集体生产留用地改变为商业服务用地，为工业园区和工业项目提供配套，增加农民收入。完成农业普查任务，为制定农业和农村发展政策提供科学依据。

　　提升村镇规划建设水平。将村镇建设纳入全市城乡一体化的整体规划。开展以改路、疏河、改厕、垃圾集中处理、村庄绿化等为主要内容的村容村貌整治，改善农村生产环境、生态环境和人居环境。加快中心镇建设，增强中心镇的产业和人口聚集功能。出台优惠政策，引导农村规划建设公寓式住宅小区，建设新型农村社区。

　　深化农村各项改革。加快农村集体资产管理体制改革和产权改革，积极稳妥推进农村股份合作制，规范土地承包经营权流转，引导发展适度规模经营。巩固农村税费改革成果，积极推进乡镇机构改革。加快红旗、平沙农场体制改革。改善农村金融服务，逐步建立现代农业保险制度。落实村民自治，大力推行村务公开、财务公开，实现集体事务民主决策、民主管理。

　　建立城乡统筹的公共服务体系。实行“工业反哺农业、城市支持农村”的方针，加强部门统筹协调，整合政策和资金，推动城市社会事业单位和公共服务向农村延伸，形成支持农村发展的合力。从今年起，市级财力用于教育、文化、卫生、体育等社会事业经费中农村所占比重要逐年递增。完善新型农村合作医疗保险制度，扩大覆盖面，适度提高筹资标准，增强保障能力。

　　（七）共享发展成果，构建社会主义和谐社会

　　千方百计做好就业工作。实施积极就业政策，加快公共就业服务体系建设。关注特困职工就业和再就业的问题，努力开发面向就业困难群体的公益性就业岗位。抓好高校毕业生、复员转业军人安置和城镇新增劳动力就业推荐工作。进一步加大劳动保障监察执法力度，依法维护劳动者的合法权益，营造稳定和谐的劳动关系。今年全市新增就业岗位要达到４．５万个以上，城镇登记失业率控制在３％以下，下岗失业人员再就业率达６０％以上，农村富余劳动力转移就业人数１万人。

　　完善多层次的社会保障体系。扩大社会保险覆盖面，提高非公有制企业从业人员的参保率。加快建立以失地农民为重点的农村保险制度，引导将土地征用收入和农村建设用地转让收入的一定比例，用于支付征地农民的养老保险和医疗保险，争取农村养老保险覆盖率达３５％以上。积极推进企业退休人员社会化管理，全面落实城乡最低生活保障制度。逐步推开未成年人医疗保险。

　　改善困难群众生活。着力解决人民群众最关心、最直接、最现实的利益问题。加快困难职工帮扶中心建设，完善社会救助体系。进一步改善城镇特困户住房状况，加快红旗、平沙、白藤农场和斗门农村危房改造。着手解决桂山岛用水用电难问题，加快海岛公路建设。制定扶助贫困残疾人实施办法，建立助残济困长效机制。建设社会福利中心和敬老院。保护进城务工人员的合法权益，努力解决外来工子女入学、劳动权益保护、法律援助等方面的实际困难。加强征地管理，落实建立征地事前公告听证和征地款预存制度，完善征地补偿收入和农村土地收入村账镇代管制度，切实保护征地农民利益。

　　维护公共安全和社会秩序。进一步强化社会治安综合治理，创新流动人口管理思路和措施，加强公安基层基础建设，科技强警，从优待警，严厉打击各种违法犯罪活动，确保治安秩序持续好转。进一步规范执法行为，促进执法公正。增强普法工作实效，切实提高群众法律意识。提高市政府投诉中心的协调能力，认真解决群众反映的问题。贯彻《信访条例》，加强信访和人民调解工作，维护社会稳定。落实安全生产责任制，严格安全生产许可证制度，加快安全生产监督执法队伍建设。开展重点行业和重点单位安全隐患专项整治行动，遏制重特大安全事故的发生。继续进行道路交通安全综合整治，压减交通事故。加强森林防火工作。

　　繁荣发展各项社会事业。优先发展教育事业。大力扶持农村教育，推动基础教育均衡化发展。从今年秋季新学年开始，全市农村义务教育阶段全部免除学杂费。加快普通高中教育发展。加大斗门创建“教育强区”工作力度，全面推进建设“教育强市”。调整中等职业教育的布局结构，大力发展中等和高等职业教育，培养大批适应新型工业化需要的高素质、技能型人才。加快广播电视发射台、古元美术馆等文化设施和基层文化站建设，加强文化遗产保护工作。深化文化体制改革，发展文化事业和文化产业，促进文化与经济融合。继续推进有线数字电视整体转换工作。加紧市人民医院、市拱北医院、市妇幼保健院改扩建工程建设，加快乡镇卫生院改造，完善农村和社区卫生服务网络，规范医院管理，提高服务水平。抓紧完善卫生规划，加快公共卫生体系建设，重视防治职业病。加强对食品、药品、化妆品和餐饮业卫生的监管，进一步加强动物疫病防控体系建设。发展体育事业和体育产业，加快市体校建设，办好国际跳水（中国站）等重大赛事。继续做好人口与计划生育工作，加快市计生服务中心改扩建工程。继续贯彻男女平等基本国策，实施妇女儿童发展规划，维护妇女儿童合法权益。加强国防教育，加大民兵预备役和国防动员工作力度，继续做好双拥工作。继续做好民族、宗教、人防、国安、对台、外事、侨务、统计、档案、仲裁、气象、老年人、残疾人、地方志和港澳流动渔民工作，支持和保障各人民团体履行职能，共同推进和谐社会建设。

　　积极化解债务。要以负责任的态度处理历史债务，正确处理发展与还债的关系，在加快发展的基础上逐步清偿债务。加强与债权人沟通，注重按照市场经济规则来化解债务。

　　（八）立足于服务为民，建设依法行政的服务型政府

　　全面推进依法行政。贯彻执行《公务员法》，进一步规范公务员管理。深入贯彻实施《行政许可法》和《全面推进依法行政实施纲要》。建立健全重大决策程序制度、事前论证和事后评估制度、决策责任追究制度，促进依法决策、民主决策和科学决策，防止重大决策失误。推行行政执法责任制，建立执行问责制，提高政府执行力。强化审计监督，建立健全对公务员履职行为有效的监督机制。

　　深入推行政务公开。整合资源，加快行政服务大厅建设，实行“一站式”服务，提高服务效能。推行指定部门代办项目制，进一步提高重点项目现场服务水平。深化行政审批制度改革，大力发展电子政务，建设网上政务服务平台。积极实施新闻发言人制度和“阳光政务”工程，加强文件阅览服务中心建设，及时向社会公开政务信息，进一步提高政府工作的透明度。

　　提高预防和处置突发事件能力。贯彻落实《珠海市突发公共事件总体应急预案》，健全应急机构，细化和落实应急工作方案，提高保障公共安全和处置突发公共事件及风险的能力。依法公开突发事件处置情况，提高应急处理工作透明度。整合１１０、１１９、１２２等应急报警资源，实现一个号码接警，快速处理各种警情和紧急求助。

　　切实加强政风建设。继续深化机关作风建设，加强行政效能监察，加强督办工作力度，整治推诿扯皮、敷衍了事、效率低下等顽症，努力形成高效、务实的工作作风。坚持抓好教育、制度、监督并重的惩治和预防腐败体系建设，坚决纠正损害群众利益的不正之风。坚决不搞“政绩工程”、“形象工程”，为民多办实事、好事，树立“为民、务实、清廉、高效”的政府形象。

　　同志们！今年的政府工作千头万绪，任务艰巨，责任重大。让我们更加紧密地团结在以胡锦涛同志为总书记的党中央周围，高举邓小平理论和“三个代表”重要思想伟大旗帜，全面落实科学发展观，认真贯彻市委五届五次全会精神，同心同德，锐意进取，真抓实干，为推动珠海经济社会发展上新台阶、开创珠海现代化建设的新局面而努力奋斗！
